# Engineering nanoparticles for macrophage reprogramming in chronic inflammatory diseases: current advances, challenges, and future perspectives

**DOI:** 10.3389/fbioe.2026.1900076

**Published:** 2026-08-11

**Authors:** Shreya Bharti, Alexander G. Obukhov, Sabahat Zafar, Luciano Saso, Mirza S. Baig

**Affiliations:** 1 Mehta Family School of Biosciences and Biomedical Engineering (MFSBSBE), Indian Institute of Technology Indore (IITI), Indore, Madhya Pradesh, India; 2 Department of Anatomy, Cell Biology and Physiology, Indiana University School of Medicine, Indianapolis, IN, United States; 3 Divisional Railway Hospital, Central Railway, Nagpur, Maharashtra, India; 4 Institute of Evolutionary Medicine, University of Zurich, Zurich, Switzerland; 5 Department of Physiology and Pharmacology “Vittorio Erspamer”, Sapienza University, Rome, Italy

**Keywords:** chronic inflammation, immunomodulation, macrophage reprogramming, nanomedicine, nanoparticles

## Abstract

Inflammation is essential for host defense and tissue repair, yet its dysregulation contributes to the development of numerous chronic diseases. Macrophages play a central role in these processes through their remarkable functional plasticity and ability to adopt pro-inflammatory or pro-resolving states. Nanoparticles have emerged as promising immunomodulatory platforms capable of reprogramming macrophage function through precisely engineered physicochemical properties. This review examines how nanoparticle characteristics, including size, surface charge, surface functionalization, biomimetic coatings, and microenvironment-responsive features, influence macrophage reprogramming across diverse chronic inflammatory diseases. Comparative analysis reveals that successful nanoplatforms frequently target conserved inflammatory pathways, including NF-κB, NLRP3, HIF-1α, and oxidative stress-associated pathways. We further highlight emerging structure-function relationships, translational challenges, and key design principles that can guide the development of next-generation nanotherapeutics aimed at restoring homeostasis in chronic inflammatory diseases.

## 1 Introduction

Inflammation is a tightly regulated biological process essential for host defense and tissue repair, yet its dysregulation is central to the pathogenesis of a wide range of chronic diseases, including cardiovascular disorders, neurodegeneration, and autoimmune conditions (Huang et al., 2024a). The molecular complexity of inflammation arises from the interplay of cytokines, chemokines, and diverse immune cell subsets, which coordinate both the initiation and resolution phases but can, under persistent activation, lead to tissue destruction and impaired organ function (Zhu et al., 2021; Huang et al., 2018). Chronic inflammation can result from ongoing exposure to harmful stimuli or failed resolution of acute inflammation, contributing to the progression of disorders (Huang et al., 2025). Persistent inflammatory responses disrupt tissue homeostasis and contribute to disease chronicity. The inflammatory microenvironment, including oxidative stress and cellular debris, plays a pivotal role in regulating macrophage function and shaping both local and systemic immune responses (Wang et al., 2021).

**FIGURE 1:**
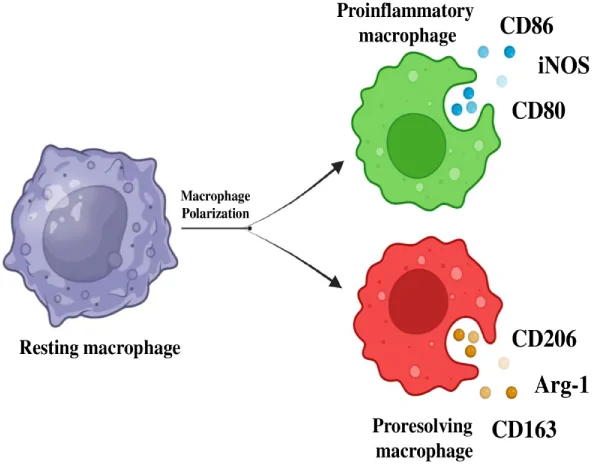
Spectrum of macrophage functional states during inflammation and resolution.

Macrophages serve as key elements of the immune response, acting as the primary agents for phagocytic clearance of foreign pathogens and materials, thereby maintaining local homeostasis in innate immunity and facilitating antigen processing in adaptive immunity (Chen et al., 2023). High-dimensional transcriptomic and system-level studies have shown that macrophage activation spans a wide, context-dependent range of states, rather than a simple binary M1/M2 model. This has led to a shift towards viewing macrophage behavior through network- or spectrum-based models (Nahrendorf and Swirski, 2016; Li. et al., 2019a; Katkar and Ghosh, 2023). Single-cell RNA-seq analyses further reveal multiple intermediate and tissue-restricted macrophage states with distinct gene-expression programs, underscoring that the classical M1 and M2 categories capture only the extremes of a far more heterogeneous landscape (Li. et al., 2019a; Saliba et al., 2016; Mould et al., 2019). Current nomenclature guidelines therefore recommend describing macrophages according to their inducing signals, transcriptional signatures, and effector functions, rather than relying solely on the historical M1/M2 types (Katkar and Ghosh, 2023; Murray et al., 2014). Within this spectrum of macrophage states, macrophages that produce high levels of pro-inflammatory cytokines can be broadly termed “pro-inflammatory” macrophages, whereas those driven by specialized pro-resolving mediators and tissue-repair pathways are better described as “proresolving” macrophages (Dalli and Serhan, 2016; Serhan et al., 2011) (Figure 1). The polarization state of macrophages determines their involvement in diverse physiological processes, including pathogen elimination and tissue regeneration. Nevertheless, when this polarization becomes imbalanced, it can contribute to the development of conditions such as cancer, tuberculosis, and various autoimmune diseases (Miao et al., 2017; Erdem et al., 2023).

Nanoparticles (NPs) represent an innovative strategy for biomedical applications and are increasingly employed in medical diagnostics, drug delivery, and tissue engineering (Subbiah et al., 2010). NPs enhance drug delivery by improving target specificity and enabling controlled release in areas with restricted drug penetration, such as tumors in the brain. They increase drug solubility, protect sensitive molecules from breakdown, and reduce drug side effects, which leads to more effective therapy and better patient tolerance, overcoming the limitations of traditional treatments (Yetisgin et al., 2020; Krol et al., 2013; Kumarasamy et al., 2024; Desai, 2012; Gelperina et al., 2005). However, NPs may induce oxidative stress and inflammation, diminishing therapeutic effectiveness and causing detrimental effects on biological systems (Buzea et al., 2007). Therefore, although recent advances in nanotechnology have revealed therapeutic promise, the potential drawbacks of nanoparticles on inflammatory pathways need to be factored into the rational design of nanomedicines (Li et al., 2020). One avenue for boosting efficacy and limiting the adverse effects of NPs is to strictly regulate their properties, such as size, shape, and surface modifications (Wang and Wang, 2014). Elucidating the connection between NP characteristics and inflammatory responses is also essential for fully exploring NPs’ potential in biomedical applications.

Interactions between nanomaterials and macrophages can lead to both acute and chronic inflammatory diseases (Lameijer et al., 2013). The extent of macrophage reprogramming by NPs can alter the inflammatory balance and has direct consequences for the resolution or perpetuation of pathological states in disorders such as rheumatoid arthritis, inflammatory bowel disease, and sepsis. Owing to their distinct physicochemical characteristics, nanoparticles engage actively with macrophages, modulating their polarization and altering functional responses (Miao et al., 2017; Xiao et al., 2023). NP stability within biological systems depends on factors such as composition, size, and surface properties. Exposure to bodily fluids leads to changes or a slow breakdown of these particles. Once administered, most NPs are rapidly eliminated from the bloodstream, usually within a few hours (Yang et al., 2018; Alexis et al., 2008; Hoshyar et al., 2016; Muraleetharan et al., 2019). Small NPs are eliminated mainly by the kidneys, whereas larger NPs are mostly phagocytosed by macrophages. Recent reviews have comprehensively discussed the role of nanoparticle physicochemical properties in regulating macrophage polarization and immunomodulatory responses, highlighting the importance of parameters such as size, surface chemistry, morphology, stiffness, and nano-bio interactions in determining macrophage fate and therapeutic efficacy (Lin et al., 2025). Furthermore, nanomaterial-driven macrophage polarization has been extensively explored as an emerging strategy for immunomodulation and regenerative medicine, with particular emphasis on structure-activity relationships and nanomaterial design principles (Dahri et al., 2025).

Certain NPs have been reported to activate the inflammasome complex within macrophages, thereby eliciting inflammatory responses (Martinon et al., 2006; Morishige et al., 2010; Reisetter et al., 2011). Notably, the surface modification of nanoparticles can significantly alter the macrophage activation pathways and the resultant immune responses. In line with this modern view, this review specifically examines nanoparticle-mediated macrophage reprogramming across diverse chronic inflammatory diseases, with a focus on disease-specific microenvironment influences, translational challenges, and shared mechanistic principles that may provide avenues for improved rational drug design.

Throughout this review, the terms “M1” and “M2” are used primarily to reflect the nomenclature adopted in the cited studies; however, we recognize that macrophage activation exists along a continuum of functional states and interpret these classifications within this broader context.

## 2 Physicochemical properties of nanoparticle governing macrophage reprogramming

The immunomodulatory effects of NPs are strongly influenced by their physicochemical characteristics including particle size, surface charge, hydrophobicity, surface functionalization, ligand density, and protein corona formation. These properties collectively regulate NP biodistribution, cellular uptake, intracellular trafficking, and macrophage activation pathways, ultimately determining macrophage polarization outcomes (Li et al., 2020; Xiao et al., 2023).

Macrophage reprogramming is closely associated with metabolic remodeling. Pro-inflammatory macrophages predominantly rely on aerobic glycolysis, which supports rapid ATP production and the synthesis of inflammatory mediators, whereas pro-resolving macrophages primarily utilize oxidative phosphorylation (OXPHOS) and fatty acid oxidation to sustain tissue repair and inflammation resolution. Consequently, nanoparticle-mediated regulation of metabolic pathways, including AMPK, PPAR- γ, and HIF-1α signaling, can shift macrophage metabolism toward a pro-resolving phenotype by suppressing glycolysis and promoting mitochondrial oxidative metabolism. These metabolic adaptations represent an important mechanism through which engineered nanoparticles modulate macrophage function across diverse inflammatory diseases (Nahrendorf and Swirski, 2016; Li. et al., 2019a; Katkar and Ghosh, 2023; Mould et al., 2019; Murray et al., 2014).

Particle size is a major determinant of macrophage interaction and tissue penetration. Smaller NPs (<50 nm) generally exhibit improved tissue penetration and prolonged intracellular retention, whereas larger particles are more readily recognized and phagocytosed by macrophages of the reticuloendothelial system (Alexis et al., 2008; Hoshyar et al., 2016). Several studies suggest that NPs ranging between 20 and 100 nm achieve optimal macrophage uptake while minimizing rapid renal clearance, thereby enhancing therapeutic efficacy in inflammatory diseases (Hoshyar et al., 2016).

Surface charge also critically affects macrophage response. Positively charged NPs often display enhanced cellular uptake because of electrostatic interactions with negatively charged cell membranes; however, excessive cationic charge may induce cytotoxicity, inflammasome activation, and oxidative stress (Buzea et al., 2007; Xiao et al., 2023). In contrast, neutral or mildly negative NPs generally exhibit improved biocompatibility and prolonged systemic circulation.

Surface modification strategies such as PEGylation, ligand conjugation, and biomimetic membrane coating further influence macrophage polarization by altering protein adsorption and immune recognition. PEGylation reduces opsonization and nonspecific phagocytosis, thereby prolonging circulation time, although repeated exposure may generate anti-PEG immune responses (Desai, 2012). Targeting ligands such as folic acid, mannose, and hyaluronic acid enhance selective uptake by inflammatory macrophages expressing corresponding surface receptors, improving site-specific immunomodulation (Hao et al., 2024; Qin et al., 2025; Zhou et al., 2021). In addition to ligand identity, ligand density on the nanoparticle surface can significantly influence macrophage recognition and uptake. Excessively low ligand density may result in insufficient receptor engagement, whereas very high densities can cause steric hinderance, altered protein adsorption, or receptor saturation. However, systemic studies evaluating the relationship between ligand density and macrophage polarization remain scarce, representing an important knowledge gap for future nanoparticle design.

Another emerging determinant is protein corona formation, where serum proteins adsorb onto NP surfaces immediately after systemic administration. The composition of the protein corona can substantially alter NP identity, macrophage recognition, inflammatory signaling, and polarization behavior (Xiao et al., 2023; Muraleetharan et al., 2019). However, current studies remain limited by inconsistent experimental conditions and lack of standardized characterization approaches, making direct comparisons between NP systems difficult. Protein corona formation also profoundly influences nanoparticle biodistribution and immune interactions by masking engineered surface properties and targeting ligands. The composition of the corona depends on nanoparticle size, surface chemistry, charge, and the surrounding biological environment, resulting in dynamic changes in biological identity after systemic administration (Xiao et al., 2023; Yang et al., 2018; Alexis et al., 2008; Hoshyar et al., 2016; Muraleetharan et al., 2019). Consequently, protein adsorption can alter macrophage recognition, cellular uptake, circulation time, and immune responses by either promoting opsonization and phagocytic clearance or reducing immune recognition (Xiao et al., 2023; Alexis et al., 2008). These effects contribute to variability in therapeutic efficacy and represent a major challenge for the clinical translation of macrophage-targeted nanomedicines. Therefore, rational control of protein corona formation through surface engineering and biomimetic strategies has emerged as an important area of future research (Lin et al., 2025; Dahri et al., 2025).

Collectively, these observations indicate that macrophage polarization is not governed by a single NP property but rather by the integrated interplay of size, charge, surface chemistry, and biological interactions. Future studies should prioritize standardized structure-function analyses and systemic comparative studies to establish predictive design rules for macrophage-targeted nanotherapeutics.

To facilitate comparison of emerging structure-function relationships, Table 1 summarizes the major physicochemical properties of nanoparticles, their effects on macrophage interactions, and their reported influence on polarization outcomes. The disease-specific examples discussed in the following sections further illustrate how these physicochemical properties are adapted to distinct inflammatory microenvironments, while a comparative structure-activity relationship (SAR) analysis is presented after the disease review to integrate these observations across different pathological conditions.

**TABLE 1:** Influence of nanoparticle physicochemical properties on macrophage polarization.

Physicochemical property	General effect on macrophages	Influence on polarization	Representative examples
Small particle size (<50 nm)	Improved tissue penetration and intracellular retention	Enhanced macrophage uptake; may favor anti-inflammatory effects depending on coating	Fe-Qur NCNs, AuNPs
Larger particle size (>100 nm)	Increased phagocytosis by RES macrophages	Higher inflammatory recognition and clearance	SPIONs, polymeric NPs
Positive surface charge	Strong cellular uptake	May enhance M1 activation and ROS generation at high charge density	PEI-coated systems
Neutral/slightly negative charge	Reduced nonspecific interactions	Improved circulation and biocompatibility	PEGylated NPs
PEGylation	Reduced opsonization and immune clearance	Prolonged circulation; lower unintended macrophage activation	Ag-CeNP@Cel
Liquid functionalization	Receptor-specific targeting	Enhanced M2 repolarization efficiency	FA-AgNPs, man-CUR NPs
Biomimetic membrane coating	Immune evasion and inflammatory homing	Improved macrophage-selective delivery	KPF@MM-NPs, MELT
ROS-responsive surfaces	Oxidative microenvironment sensitivity	Suppression of NF-κB/NLRP3 signaling and promotion of M2 phenotype	PFTU@DEX, BSA-BR-Pt
Protein corona formation	Alters biological identity	Modify macrophage activation	General nanomaterials

Abbreviations: Ag-CeNP@Cel: Siver-cerium nanoparticle-celastrol nanoplatform, AuNPs: Gold nanoparticle, BSA-BR-Pt: Bovine serum albumin-bilirubin-platinum nanoparticles, FA-AgNPs: Folic acid-functionalized silver nanoparticles, Fe-Qur NCNs: Iron-quercetin nanocoordination networks, KPF@MM-NPs: Kaempferol-loaded macrophage membrane-coated nanoparticles, Man-CUR NPs: Mannose-functionalized curcumin nanoparticles, MELT: Macrophage membrane-coated PLGA, nanoparticles, PEG: polyethylene glycol, PEI: polyethyleneimine, PFTU@DEX: ROS-responsive dexamethasone-loaded polyurethane thioketal nanoparticles, ROS: reactive oxygen species, SPIONs: Superparamagnetic iron oxide nanoparticles.

Having established the universal design principles governing nanoparticle-mediated macrophage reprogramming, the following disease-specific sections illustrate how these common engineering strategies-including particle size optimization, surface functionalization, biomimetic coatings, ligand-mediated targeting, ROS responsiveness, and controlled drug release-are tailored to distinct pathological microenvironments. Rather than representing isolated case studies, these examples demonstrate how shared nanoparticle engineering principles are adapted to meet disease-specific therapeutic requirements, while converging on conserved mechanisms of macrophage reprogramming.

1. **Rheumatoid Arthritis (RA):** RA is the most prevalent form of chronic inflammatory arthritis and affects ∼1% of the global population, causing persistent synovial inflammation and cartilage deterioration. Bovine serum albumin-bilirubin-platinum NPs (BSA-BR-Pt NPs) serve as a nanoplatform that alleviates hypoxia and scavenges reactive oxygen species (ROS) for the treatment of RA. RNA sequencing and experiments showed these NPs repolarize hypoxic M1 macrophages to M2 by inhibiting the HIF-1α pathway, shifting metabolism from glycolysis to oxidative phosphorylation (Guo et al., 2024). A cyanobacteria-based micro-nanodevice was also created for controlled oxygen release in RA joints to suppress HIF-1α, reducing inflammation by promoting macrophage reprogramming towards pro-resolving functional states. Although many of the cited studies describe this transition as M1-to M2 polarization, these phenotypes represent the extremes of a broader activation spectrum (Guo et al., 2021).

Ultrasmall iron-quercetin coordination NPs (Fe-Qur NCNs) have recently emerged for RA treatment, displaying potent anti-inflammatory and antioxidant effects. In-vitro studies confirmed that they clear ROS, block apoptosis, and curb inflammatory macrophage polarization by inhibiting NF-κB activation (Han et al., 2023a). Folic acid-modified silver NPs (FA-AgNPs) target M1 macrophages to reduce their level, induce apoptosis, and drive M1-to-M2 repolarization, marking the first such dual action RA therapy (Yang et al., 2021). Zhou et al. showed Fum-PD (Fumagillin prodrug) nanotherapy suppressed inflammation in RA mouse models; *in vitro*, it activated AMPK to foster anti-inflammatory M2 phenotypes and reduce M1 cytokine production (Zhou et al., 2014).

The Ag-CeNP@Cel nanoplatform features an Ag-CeNP core, PEG (polyethylene glycol) coating, and celastrol (Cel) loading (Zhang et al., 2025a), enabling robust ROS scavenging, reducing pro-inflammatory signals, and enhancing anti-inflammatory responses for M1-to-M2 macrophage switching (Yang et al., 2021; Veronesi et al., 2015). Biomimetic RM/MTX/AMM micelles (pH-responsive micelles of mPEG-PAsp-IM with methotrexate/LPS-activated macrophage microvesicles) dual-target inflamed tissues and M1 macrophages, driving M1-to-M2 reprograming (Hao et al., 2024). HSA-based pDNA/DSP NPs (chemotherapeutic drug dexamethasone sodium phosphate), made via desolvation, extend blood circulation, target inflamed sites, and outperform single-drug NPs in repolarizing M1 to M2 macrophages (Zheng et al., 2022). Hyaluronic acid-coated Fe-MOF NPs co-deliver emodin and siKEAP1, binding CD44 on M1 macrophages to reduce ROS/inflammation and promote M2 polarization (Qin et al., 2025).

TP-loaded folic acid-liposomes (FA-Lips-NPs) reduce M1 levels, repolarize to M2, and alleviate joint inflammation for targeted RA therapy (Zhou et al., 2021). A DNA-based co-assembly platform with folic acid-functionalized NPs (via 5′-amino DNA-NHS-FA conjugation) enhances joint drug retention and targets macrophages post-injection, amplifies antioxidants, and reprograms M1 to M2 polarization to halt RA progression (Tian et al., 2025). Among the reported platforms, FA-AgNPs appear particularly promising because they combine M1 macrophage apoptosis with macrophage repolarization. However, direct head-to-head comparisons among different NP systems are lacking, making definitive conclusions regarding relative efficacy difficult. These differences stem from targeted delivery and multifaceted mechanisms, with biomimetic platforms (Hao et al., 2024; Tian et al., 2025) showing promise for translation due to enhanced retention.

Collectively, studies in RA suggest that therapeutic efficacy depends not only on the anti-inflammatory cargo but also on nanoparticle features that enhance synovial accumulation, hypoxia responsiveness, and macrophage-specific targeting. However, the relative contribution of these design parameters remains unclear, highlighting the need for comparative studies that establish predictive structure-function relationships and facilitate clinical translation.

2. **Periodontitis:** Periodontitis, a common chronic inflammatory disease of the gums, destroys tooth-supporting tissues via dysregulated immune response to dental biofilms (Łasica et al., 2024). L-cysteine-caps 45 nm gold NPs (AuNPs) for stability via gold-thiol bonds, optimizing surface charge, biocompatibility, and macrophage uptake. This modification allows AuNPs to reshape the immune microenvironment by blocking M1 macrophage polarization, lowering iNOS and pro-inflammatory cytokines, and fostering periodontal tissue repair *in vitro* and *in vivo* through pathways like tuberin-mTOR/NF-κB, p38 MAPK/NF-κB, and AP-1 (50). Polyethylene glycol-coated gold NPs (GNPs) curb bacterial infection-triggered inflammation in macrophages. PEG coating improves gold NPs’ biocompatibility, preventing pro-inflammatory cytokine induction while enabling non-cytotoxic inhibition of LPS-driven inflammation in macrophages via TRIF/MyD88 pathways: they suppress STAT1 phosphorylation by decreasing IFN-β in the TRIF arm and block NF-κB via Akt inhibition, preventing IkB-α degradation in the MyD88 arm, ultimately reducing iNOS expression, NO output, and M1 polarization (Ma et al., 2010). Resveratrol-loaded ginsenoside (PPD) NPs (RES@PPD NPs) block M1 polarization, drive M2 shifts, clear excess ROS, curb local inflammation, and mitigate the progression of periodontitis in rat models (Huangfu et al., 2023).

Researchers investigated the therapeutic effects of G3@SeHANs and PAMAM-G3 NPs, which neutralize cf-DNA to dampen pro-inflammatory responses *in vitro* and limit inflammation-induced bone loss in ligature-induced mouse periodontitis models. G3@SeHANs excelled at promoting M2 macrophage polarization and outperformed PAMAM-G3 in reducing inflammation and alveolar bone resorption (Wang et al., 2024). Zeolitic imidazole framework-8 (ZIF-8) forms from ZN^2+^ and imidazole ligands, enabling controlled ZN^2+^ release for osteogenesis and antibacterial action in biomedicine (Abdelhamid, 2021). ZIF-8 doped with 10% cerium (ZIF-8:Ce10%) enhances anti-inflammatory effects by halting NF-kB/p65 nuclear translocation, favoring M2 macrophage polarization (Li X. et al., 2019). A bifunctional polymer vesicle nanoplatform (PV/Ag@IL-4), self-assembled from polystyrene-block-polyacrylic acid with silver NPs and IL-4, restores periodontal bone balance by resolving inflammation. Local gingival delivery *in vivo* reduced alveolar bone loss, advanced M2 macrophage polarization, optimized the oral immune environment, and improved periodontitis treatment (Li et al., 2024).

G3@SeHANs demonstrated enhanced cf-DNA neutralization and M2 polarization compared with PAMAM-G3 under the reported experimental conditions. However broader comparisons across nanoparticle platforms remain limited.

Collectively, periodontitis-associated nanoplatforms indicate that both ROS-scavenging and immune-directing approaches can promote macrophage repolarization. However, systems integrating antimicrobial activity with active M2-promoting signals may provide broader control over the inflammatory microenvironment characteristics of biofilm-driven disease.

3. **Acute Myocardial Infarction (AMI):** AMI, a top global cause of death and heart failure, involves monocyte-driven inflammation that worsens ischemia-reperfusion (IR) injury and impairs post-AMI healing. Pioglitazone NPs, showing PPARγ agonism, curb monocyte/macrophage inflammation. In mouse MI models, 3-day treatment starting 6 h post-left anterior descending artery ligation drove M2 polarization via NF-κB inhibition, reduced cardiac remodeling, lowered mortality, and eased IR injury (Tokutome et al., 2019). Calcium carbonate NPs (ColCaNPs) deliver colchicine to combat AMI-related morbidity, where post-ischemic inflammation and pyroptosis fuel fibrosis, remodelling, and heart failure (Wang et al., 2022; Ni et al., 2019). ColCaNPs favor M2 over M1 polarization; Liu et al. showed TLR4/NF-κB inhibition regulates this shift and enhances cardiac function post-MI (Liu et al., 2019). Similarly, Wang Y. found that low-dose colchicine curbs caspase-1, enhances M2 markers, and limits IL-1β (Wang et al., 2016). Overall, ColCaNPs block TLR4/NF-κB/NLRP3 signaling for acute-phase anti-inflammatory and cardioprotective benefits.

Mannan-coated metal-organic framework NPs (Que@MOF/Man) target quercetin to inflamed MI tissue, shifting macrophages from pro-inflammatory M1 to reparative M2 phenotypes to resolve inflammation and support cardiac repair. They modulate NF-κB, TNF, IL-17, JAK-STAT, and MAPK pathways, fostering regenerative macrophage activity, curbing inflammation, and shielding cardiomycetes (Hu et al., 2024). PLGA NPs (TAK-242-NPs) carry TLR4 inhibitor TAK-242 to treat AMI IR injury, blocking MyD88/TRIF adaptor binding, downstream IRAK4-driven NF-κB/MAPK (JNK/p38) activation, myocardial IL-6/MCP-1 expression, and inflammatory cytokine release (Fujiwara et al., 2019).

Collectively, these studies suggest that AMI-targeted nano-therapies operate through complementary mechanisms rather than a clear hierarchy of efficacy. PPARγ agonist-loaded systems primarily support long-term cardiac remodeling and inflammation resolution, whereas colchicine and TLR4-targeted platforms appear particularly effective during the acute inflammatory phase. This highlights the importance of matching nanoparticle design to the temporal dynamics of post-infarction inflammation.

4. **Atherosclerosis (AS):** AS, a leading cause of death globally, is characterized by chronic macrophage inflammation from initiation to complications of the arterial intima driven by lipids. Re-polarizing M1 macrophages to M2 macrophages could be an effective strategy for treating AS. Studies revealed kaempferol (KPF) as a promising anti-inflammatory agent. To enhance its therapeutic potential, a macrophage-biomimetic KPF delivery system, termed KPF@MM-NPs was developed. These ROS-responsive dextran-g-PBMEO NPs were coated with macrophage membranes (MM) to target and accumulate in atherosclerotic lesions. Treatment with KPF@MM-NPs significantly reduced macrophage inflammation, lowered key pro-inflammatory cytokines, and promoted M1 to M2 polarization via inhibition of the NF-κB pathway, leading to substantial anti-AS effects in ApoE−/− mice after intraperitoneal delivery (Zhao et al., 2023). cRGD-platelet@MnO/MSN@PPARα/LXRα NPs are novel NPs designed to treat AS and evaluate their mechanism for inhibiting inflammation and reducing lipid levels. These NPs significantly reduced plaque formation, improved lipid profiles by lowering LDL-cholesterol, total cholesterol, and triglycerides, while increasing HDL-cholesterol levels. They also decreased serum and aortic tissue inflammatory markers, indicating reduced inflammation and promoting M2 macrophage polarization by suppressing NF-κB signaling (Lv et al., 2025). HDL mimetic nanotherapeutics (High-density lipoprotein mimetic NPs) show promise for addressing cardiovascular issues by modulating interactions between monocyte-derived cells and HDL mimetics, thereby influencing inflammation, lipid metabolism, and atherosclerotic plaque progression. Research indicates that these nanotherapeutics can regulate monocyte recruitment and promote macrophage polarization towards an anti-inflammatory phenotype, potentially slowing atherosclerosis development (Zhen et al., 2024). The bioactivatable PROTAC system comprises PLGA-NPs loaded with the PROTAC degrader dTRIM24 and coated with M2 macrophage membranes (MELT). *In vitro* analyses, including RT-qPCR, Western blot, and FACS, confirmed that MELT induced TRIM24 degradation and shifted M1 macrophages toward the M2 phenotype by inhibiting NF-κB and MMP activity by suppressing the EMMPRIN/MMP pathway. Evaluation of atherosclerotic plaques by Oil Red O staining showed a significant decrease in lipid accumulation in the aorta and aortic roots (Ma et al., 2024). Neutrophil membrane-coated P5c polypyridine NPs deliver superoxide dismutase (SOD) and catalase (CAT) to macrophages, inducing autophagy via reduced ROS and blocked foam cell formation. They drive M2 polarization, eliminate plaque senescent cells, and restore spleen structure in ApoE−/− mice (Bartusik-Aebi et al., 2025).

The various NP platforms evaluated in atherosclerosis appear to address distinct pathological features. For example, KPF@MM-NPs primarily target inflammatory signaling, cRGD-platelet@MnO/MSN@PPARα/LXRα NPs combine immunomodulation with lipid regulation, whereas P5c polypyridine NPs additionally target oxidative stress and cellular senescence. These observations suggest that multifunctional platforms may offer broader therapeutic benefits; however direct comparative studies are needed to determine their relative clinical potential.

Across atherosclerosis studies, successful nanotherapeutics commonly target inflammatory macrophages while simultaneously modulating lipid metabolism and oxidative stress pathways. Nevertheless, long-term safety, plaque-specific delivery efficiency, and scalability of multifunctional nanoparticle systems remain important challenges that must be addressed before clinical implementation. Rather than representing a strict transition between two discrete phenotypes, these nanoparticle-mediated changes likely reflect progressive remodeling of macrophage functional states within the complex atherosclerotic plaque microenvironment.

5. **Osteoarthritis (OA):** OA, a chronic disorder driven by inflammation, relies heavily on M1 macrophages for disease progression; shifting them to M2 offers a viable therapeutic avenue. Activated macrophages readily uptake opsonized NPs from circulation, directing them to the reticuloendothelial system (RES) organs. *In vivo*, IgG/Bb@BRPL (an opsonized nanoparticle) outperformed non-opsonized versions by targeting M1 macrophages, scavenging ROS, deactivating NF-κB, curbing inflammation, driving M2 polarization, and aiding cartilage repair, without prolonging retention (Kou et al., 2022). Polyethyleneimine (PEI)-functionalized diselenide-bridged mesoporous silica nanoparticles (MSN-PEI) are advanced nanoparticles that excel at binding cell-free DNA (cfDNA) and neutralizing ROS, key OA inflammatory mediators. They suppress M1 polarization, decrease the activation of the TLR9 signaling from cfDNA, ease oxidative stress, and dampen joint inflammation, positioning MSN-PEI as an osteo-immunomodulator to mitigate OA progression (Shi et al., 2023).

IgG/BRJ-NP combines bilirubin (BR; antioxidant/immunomodulator), JPH203 (J; mTOR inhibitor), and IgG for enhanced M1 macrophage uptake under oxidative stress. Intracellularly, BRJ dissociates to clear ROS, block NF-κB/mTOR pathways, and enable M1-to-M2 repolarization. In rat OA models, IgG/BRJ reduces pro-inflammatory markers, supports cartilage recovery, and has the potential to be a promising therapeutic agent (Huang H. et al., 2024). Host immune responses, especially macrophage inflammation, often impair bone regeneration around endosseous implants. Ag@TiO2-NTs (antibacterial silver nanoparticle-loaded titanium dioxide nanotubes containing an ultra-low dose of silver ions) create an osteo-immune balance by inhibiting M1 polarization via PI3K/Akt/GLUT1 suppression and autophagy induction, while controlled Ag + release reduces oxidative stress, drives M2 shifts, provides antibacterial effects, and enhances bone healing *in vitro* and *in vivo* (Chen et al., 2020).

IgG/BRJ-NP showed promising therapeutic effects by simultaneously targeting oxidative stress and mTOR signaling. Together with opsonized IgG/Bb@BRPL and MSN-PEI systems, these findings suggest that combining targeted macrophage uptake with multi-pathway modulation may improve therapeutic outcomes in OA.

6. **Ulcerative Colitis (UC) and Inflammatory Bowel Disease (IBD):** UC is a chronic inflammatory disease marked by persistent colon inflammation and immune system imbalance, often driven by disrupted gut barrier function and bacterial interactions (Velikova and Xiang, 2025). PSB@NP-FA is a ROS-responsive, folic acid-functionalized, poly (lactic-co-glycolic acid) (PLGA)-based NP designed for the targeted delivery of pterostilbene (PSB) to inflamed colonic tissue for the treatment of UC. PSB@NP-FA alleviates inflammation in UC by promoting macrophage polarization from the M1 to the M2 phenotype, as evidenced by *in vitro* studies. Mechanistic studies indicated that it mitigated colitis by regulating dendritic cells (DCs), promoting macrophage polarization, and modulating T-cell infiltration, involving both innate and adaptive immunity. In the dextran sulfate sodium (DSS)-induced colitis model, PSB@NP-FA demonstrated significant ROS-scavenging properties, effectively reducing colonic inflammation by modulating cellular pathways (Yan et al., 2023). Man-CUR NPs are supramolecular NPs designed for the treatment of UC, incorporating curcumin (CUR) as the therapeutic agent and utilizing β-cyclodextrin (β-CD) and D-mannose (D-Man) ligands to target macrophages specifically to effectively reduce inflammation by scavenging excess ROS. Man-CUR NPs play a crucial role in macrophage polarization by downregulating pro-inflammatory factors, upregulating anti-inflammatory factors, and promoting the shift from M1 to M2 phenotype, which is regulated through the TLR4/NF-κB signaling pathway (Han X. et al., 2023). IBD is a persistent and recurring inflammatory condition affecting the gastrointestinal tract, including ulcerative colitis (UC) and Crohn’s disease (CD). It arises from complex interactions among genetic, immune, and environmental influences. In this context, LBL-CO@MPDA (an orally administered nanotherapeutic with carbon monoxide) was designed as a nanotherapeutic strategy for the treatment of IBD; this formulation delivers carbon monoxide (CO) for therapeutic effects. CO@MPDA improves inflammatory conditions through MPDA-mediated ROS scavenging and CO-mediated immune modulation. The release of CO activates heme oxygenase-1, promoting macrophage M2 polarization via the Notch/Hes1/Stat3 signaling pathway, while simultaneously inhibiting the inflammatory response by down-regulating the p38 MAPK and NF-κB (p50/p65) pathways. Notably, through MPDA’s ROS-scavenging and CO-driven macrophage phenotype shift from M1 to M2, LBL-CO@MPDA can significantly reduce chronic inflammation by reversing the pro-inflammatory microenvironment, providing immunomodulatory effects, ultimately restoring the intestinal barrier, and modifying the gut microbiota (Zhang et al., 2023). LBL-CO@MPDA provides an example of a multifunctional platform that combines ROS scavenging, immune modulation, and microbiota-associated effects. Compared with ligand-targeted systems such as PSB@NP-FA and Man-CUR NPs, it highlights the potential advantages of integrating multiple therapeutic mechanisms within a single nanoplatform.
7. **Sepsis:** Sepsis is a life-threatening condition characterized by organ dysfunction resulting from an uncontrolled host response to systemic infection, regardless of the causative agent (Singer et al., 2016). The liver plays a central role by clearing pathogens and toxins, yet it remains highly susceptible to injury (Xu et al., 2019). Bacterial sepsis poses a major global health burden and is the leading cause among critically ill patients. Central to its pathology is a pro-inflammatory and pro-resolving immune imbalance, where either hyper- or hypo-responses can be detrimental to patient outcomes (Doi et al., 2009; Ondee et al., 2017).

Gold NPs (AuNPs) possess several biochemical advantages as potential drug carriers. Interestingly, non-conjugated AuNPs exhibit higher cellular uptake than conjugated counterparts. In CLP models, AuNP administration enhanced survival, reduced blood bacterial load, improved kidney and liver function, regulated cytokine levels and promoted macrophage polarization toward M2. This adjuvant therapy, combined with appropriate antibiotics, effectively alleviated CLP-induced bacterial sepsis in mice (Taratummarat et al., 2018). Iron-oxide-based synthetic NPs (SPIONs) have antibacterial properties and modulate inflammation. They trigger macrophage autophagy through the Cav1-Notch1/HES1 pathway, elevating IL-10 and easing liver damage in LPS sepsis models (Xu et al., 2019). Recent studies demonstrate that mesenchymal stem cells (MSCs) labelled or pretreated with SPIONs potently shift macrophages to M2 via TRAF1, while boosting MSC survival through HO-1 upregulation (Xu et al., 2021). AuNPs and SPION-based systems appear to provide complementary therapeutic benefits in sepsis. Whereas AuNPs showed broad antibacterial and immunomodulatory effects, SPION-based approaches emphasized macrophage autophagy and tissue-protective pathways. These differences highlight the importance of matching nanoparticle design to the stage and immune status of sepsis.

Unlike chronic inflammatory disorders, sepsis requires precise temporal regulation of macrophage responses to balance pathogen clearance and immune resolution. Consequently, future nanoparticles strategies should prioritize context-dependent immune modulation rather than uniform suppression of inflammatory signaling.

8. **Severe acute pancreatitis (SAP):** SAP is characterized by extensive pancreatic tissue necrosis and a systemic inflammatory response that frequently leads to multi-organ failure and high mortality, driven by premature activation of pancreatic enzymes and cytokine release (Beger and Rau, 2007). Inflammation of the pancreas is a hallmark of SAP, which often leads to multiple complications, but it is a challenge to treat this condition (Zhou et al., 2003; Granger and Remick, 2005). Macrophages play a critical role in both the progression and resolution of SAP. Emodin, a compound derived from traditional Chinese herbs, has been shown to exhibit anti-inflammatory properties and may help alleviate SAP-related inflammation. Recently, nanocapsules have been developed to enhance the bioavailability of emodin and to facilitate its controlled release for targeting macrophages. The mannose-conjugated chitosan-coated lipid nanocapsules loaded with emodin (M-CS-E-LNC) were evaluated for the treatment of SAP. M-CS-E-LNC significantly reduced the production of inflammatory mediators by cultured macrophages. Mechanistically, M-CS-E-LNC upregulated the expression of carnitine palmitoyltransferase 1 (CPT1), promoting intracellular transport of long-chain fatty acids and downregulating pro-inflammatory factors, which facilitated M2 macrophage polarization through lipid metabolic reprogramming (Song et al., 2024).

M-CS-E-LNC nanocapsules demonstrate the therapeutic potential of metabolic reprogramming for promoting macrophage repolarization in SAP. Their efficacy suggests that targeting lipid metabolism may complement more conventional antioxidant-based approaches.

9. **Acute Lung Injury (ALI):** ALI is characterized by a rapid onset of alveolar-capillary barrier disruption, resulting in pulmonary edema, excessive neutrophil infiltration, and overwhelming inflammation that impairs gas exchange and lung function (Mokra, 2021). PFTU-NP is a ROS-responsive NP that is specifically activated in tissues with elevated ROS due to cellular oxidative stress. The chemical structure of PFTU contains reactive double bonds that allow attachment of various functional substances, such as anti-inflammatory drugs. The PFTU@DEX NP scavenges excessive ROS, thereby inhibiting NLRP3 inflammasome activation. It induces macrophage polarization towards the proresolving macrophages and decreases the population of pro-inflammatory macrophages. In a study by Muhammad et al. (2022), this NP was used to inhibit the secretion of inflammatory mediators in the lungs, thereby reducing lung damage in cases of ALI (87). Macrophage activation and polarization are crucial in both triggering and resolving inflammation in ALI. Another study developed a bioactive nanodevice composed of hexapeptides and gold NPs (GNP-P12) that targeted lung macrophages after intratracheal delivery. These peptide-coated GNPs reduced macrophage activation and facilitated the M1 to M2 transition in cultured BMDM and in the lungs of ALI mice by inhibiting NF-κB activation (Wan et al., 2020). The comparison between PFTU@DEX and GNP-P12 highlights two distinct therapeutic strategies: ROS-responsive drug release and direct NF-κB inhibition. These findings suggest that microenvironment-responsive systems may offer advantages in diseases characterized by pronounced oxidative stress.
10. **Wound Healing:** Wound healing is a complex process involving various cell types, crucial for restoring the skin’s barrier function (Gurtner et al., 2008). Impaired fibroblast function, bacterial proliferation, and prolonged inflammation are primary causes of delayed healing and excessive scar formation (Morris et al., 2014; Nanditha and Kumar, 2022; Shams et al., 2022). Such delays and scars compromise aesthetics and joint mobility and affect patients’ quality of life. Gallium-modified gelatin nanoparticles loaded with quercetin (QCT@GNP-Ga) were developed for wound healing, demonstrating excellent biocompatibility and beneficial effects. They drive macrophage repolarization from M1 to M2 via the TGF-β/Smad pathway, curbing inflammation. *In vivo*, these NPs enhanced wound repair and suppressed scarring (Yang et al., 2023).

Amphiphilic polymeric NPs, such as polymeric micelles, self-assemble from copolymeric amphiphiles in water when their concentration exceeds the critical aggregation concentration (Kaga et al., 2017; Li et al., 2018). Drug-free amphiphilic NPs (hGM-g-PMMA NPs), formed by self-assembly of hydrolyzed galactomannan–a galactose-mannose polysaccharide–grafted with poly (methyl methacrylate) chains, enable drug loading. They access macrophages through clathrin–and caveolin-mediated endocytosis, shifting them from M1 to M2, with downregulated CD80 and upregulated CD163/CD206, as verified by flow cytometry and immunofluorescence (Peled and Sosnik, 2021).

Layer-by-layer nanocarriers (PEI-LBL-NC) delivering IRF5-siRNA reprogram M1 macrophages into M2 macrophages for diabetic wound healing. Chitosan-coated versions showed poor endosomal escape and transfection, but PEI-coated ones exhibited enhanced stability, uptake, transfection efficiency, and accelerated wound closure (Sharifiaghdam et al., 2021). Collectively, wound healing nanoplatforms indicate that both genetic reprogramming and immunomodulatory biomaterials can effectively promote macrophage-mediated tissue repair. The relative advantages of these approaches remain to be established through standardized comparative studies.

11. **Myocarditis:** Myocarditis, an inflammatory condition of the heart muscle, raises the risk of dilated cardiomyopathy and heart failure. Macrophage infiltration is a key pathological feature, making them a valuable therapeutic target (Muhammad et al., 2022). A study showed that a bioinspired anti-inflammatory nanomedicine, conjugated to protein-G (PSL-G), was developed to selectively target macrophages and promote their shift towards the M2 phenotype. PSL-G NP showed greater affinity for macrophages over other cells and effectively reduced pro-inflammatory cytokines (IL-1, IL-6, TNFα) while enhancing the anti-inflammatory cytokine IL-10 in macrophages (Toita et al., 2021).
12. **Uveitis, Acute Kidney Injury, Parkinson’s Disease:** Uveitis is a group of intraocular inflammatory disorders marked by uncontrolled autoimmune responses and oxidative stress, leading to vision loss. Polyvinylpyrrolidone-curcumin nanoparticles (PVP-CUR) are formed by conjugating curcumin (CUR) with polyvinylpyrrolidone (PVP), significantly enhancing solubility and radical scavenging abilities in uveitis. *In vitro* studies demonstrated that PVP-CUR-NPs effectively reduced oxidative stress and apoptosis in H2O2-induced human retinal pigment epithelial cells (ARPE-19) and promoted the shift from M1 to M2 polarization in LPS-induced human microglial cells (HMC3). PVP-CUR nanoparticles have also been reported to treat acute kidney injury (AKI) and Parkinson’s disease (PD) due to their enhanced solubility and excellent ROS scavenging properties. Their oxidoreductase-like activities, particularly superoxide dismutase (SOD) and catalase (CAT), contribute to reduced ROS levels. These findings highlight the potential therapeutic effects of PVP-CUR-NPs in uveitis and other conditions related to oxidative stress and inflammation (Cao et al., 2024).
13. **Anti-inflammation and macrophage-related disorders:** Nanomaterials with surface modifications can either enhance or suppress immune response, which is crucial for their biomedical effectiveness. In particular, changes in surface charge, hydrophilicity, and ligand density determine how NPs interact with serum proteins and are recognized by macrophage receptors. Beyond simply lowering TNF-α and IL-1β secretion, biodegradable polyurethane nanoparticles (PU NPs) downregulate NLRP3 inflammasome signaling and NF-κB activity via autophagy induction, thereby restraining M1 polarization and favoring a proresolving state. PU NPs act as active immunomodulators, reshaping the intracellular signaling environment rather than serving as inert carriers. These findings align with broader evidence that surface chemistry, nano-roughness, and polymer composition can be engineered to tune macrophage responses and mitigate the fibrotic foreign body reaction, providing a mechanistic basis for using PU-like platforms as low-FBR, immunomodulatory scaffolds in inflammatory and implant-associated disorders (Morris et al., 2014; Dabare et al., 2023).

**FIGURE 2:**
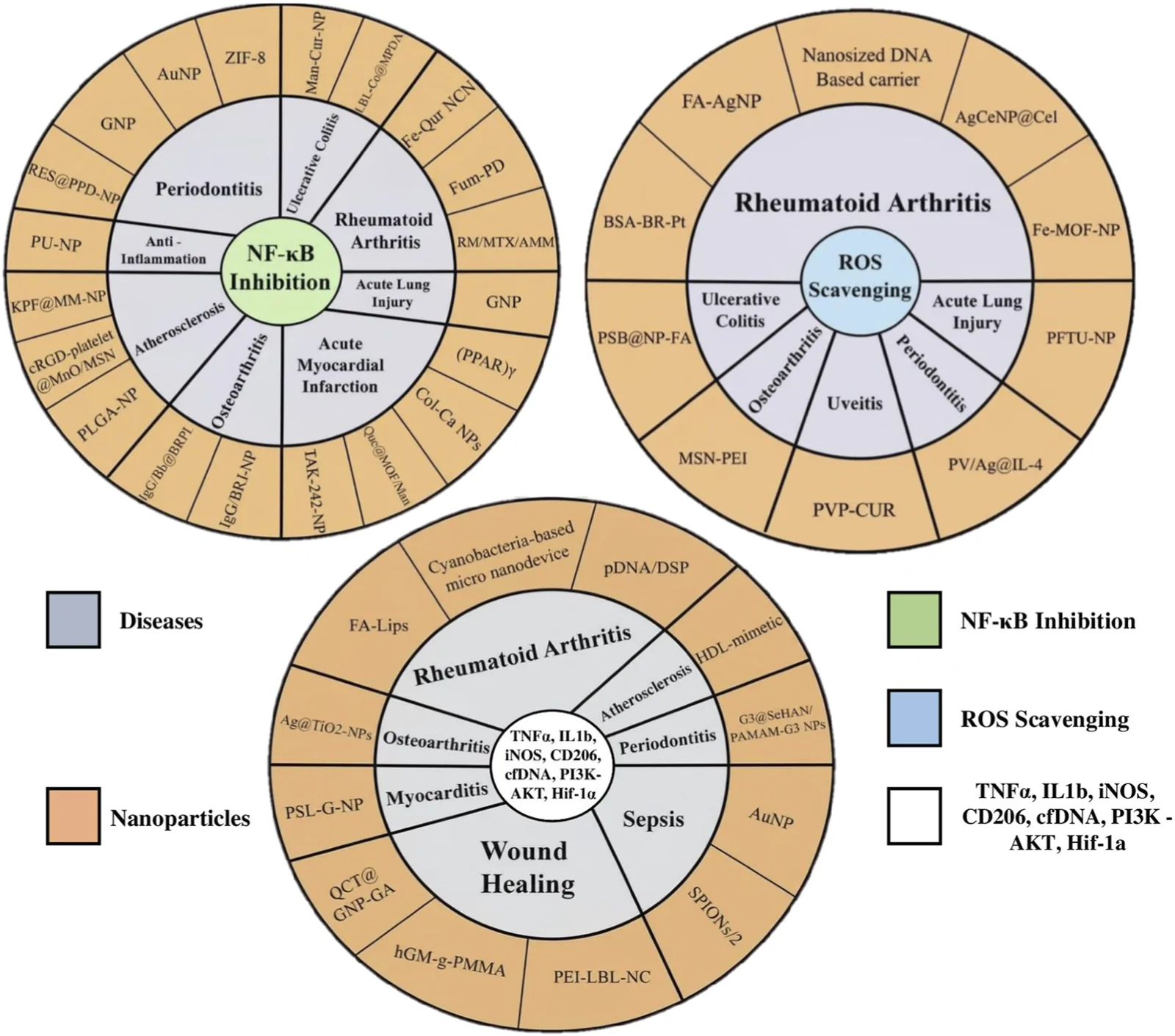
Major signaling pathways regulated by nanoparticle-mediated macrophage reprogramming across chronic inflammatory diseases. Different nanoparticle platforms modulate conserved inflammatory pathways, including NF-κB, NLRP3 inflammasome, HIF-1α, PI3K/Akt, MAPK, STAT, and oxidative stress-associated signaling, thereby promoting context-dependent macrophage reprogramming, suppressing inflammation, and improving disease-specific therapeutic outcomes.

All NPs along with disease and their mechanism of actions have been summarized in Figure 2 and Table 2.

**TABLE 2:** Tabular representation of NP category and macrophage repolarization state.

S. No.	Inflammatory disorder	Nanoparticle	Physicochemical/surface feature	Targeting strategy	Dominant mechanism	Macrophage polarization	Clinical trial	Therapeutic outcome	References
1	Rheumatoid Arthritis (RA)	BSA-BR-Pt NPs	Antioxidant bilirubin-based nanoplatform	Hypoxic inflammatory microenvironment targeting	HIF-1α inhibition and metabolic reprogramming	M1 to M2	Preclinical	Reduced synovial inflammation	Guo et al. (2024)
		Fe-Qur NCNs	Ultrasmall-iron quercetin nanocomplex	Passive macrophage uptake	ROS scavenging and NF-κB inhibition	Promote M2	Preclinical	Attenuated oxidative stress and joint damage	Han et al. (2023a)
		FA-AgNPs	Folic acid functionalized silver nanoparticle	Folate receptor mediated targeting	M1 apoptosis and M2 repolarization	M1 to M2	Preclinical	Enhanced macrophage repolarization and arthritis remission	Yang et al. (2021)
		Fum-PD PFC NPs	Fumagillin prodrug-loaded nanotherapy	Passive inflammatory tissue accumulation	AMPK activation and anti-inflammatory polarization	M1 to M2	Preclinical	Suppressed inflammatory responses in RA	Zhou et al. (2014)
		Ag-CeNP@Cel	PEG-coated cerium-containing ROS-responsive nanoplatform	ROS-responsive inflammatory targeting	ROS scavenging and NF-κB suppression	M1 to M2	Preclinical	Reduced cartilage destruction and inflammation	Yang et al. (2021), Zhang et al. (2025a), Veronesi et al. (2015)
		RM/MTX/AMM	Biomimetic macrophage membrane-coated micelles	Dual inflammatory tissue and macrophage targeting	Macrophage reprogramming	M1 to M2	Preclinical	Improved anti-inflammatory efficacy in RA	Hao et al. (2024)
		HSA-based pDNA/DSP NPs	Albumin-based biomimetic nanoparticle	Enhanced inflamed tissue retention	Anti-inflammatory macrophage repolarization	M1 to M2	Preclinical	Reduced inflammatory burden and promoted immune regulation	Zheng et al. (2022)
		Fe-MOF NPs	Fe-based metal-organic framework	CD44-mediated macrophage targeting	ROS reduction and M2 polarization	Promote M2	Preclinical	Alleviated arthritis progression and oxidative injury	Qin et al. (2025)
		FA-lips NPs	Folic acid-functionalized liposomes	Folate receptor targeting	Targeted macrophage repolarization	M1 to M2	Preclinical	Enhanced targeted anti-arthritic effects	Zhou et al. (2021)
		DNA-based NCs	Nanosized DNA carriers	Joint macrophage targeting	Antioxidant amplification and M2 polarization	M1 to M2	Preclinical	Reduced inflammation and promoted tissue protection	Tian et al. (2025)
		Cyanobacteria- micro-nanodevice	Oxygen-generating Cyanobacterial micro-nanodevice	Hypoxic tissue-responsive delivery	HIF-1α suppression and ROS reduction	Promote M2, suppress M1	Preclinical	Relieved hypoxia and inflammatory damage	Guo et al. (2021)
2	Periodontitis	AuNPs	Gold Nanoparticle	Passive macrophage uptake	NF-κB/p38 MAPK inhibition	M1 to M2	Preclinical	Reduced periodontal inflammation and bone loss	Ni et al. (2019), Ma et al. (2010)
		RES@PPD	Resveratrol Nanoparticle	ROS-responsive inflammatory targeting	ROS scavenging and M2 polarization	M1 to M2	Preclinical	Improved periodontal tissue regeneration	Huangfu et al. (2023)
		G3@SeHAN	Selenium-doped hydroxyapatite nanoparticle	Bone inflammatory micro-environment targeting	cf-DNA neutralization and M2 polarization	M1 to M2	Preclinical	Enhanced alveolar bone preservation	Wang et al. (2024)
		PAMAM-G3	Cationic poly-amidoamine dendrimers	Electrostatic inflammatory targeting	cf-DNA neutralization	M1 to M2	Preclinical	Reduced cfDNA-mediated inflammation	Wang et al. (2024)
		ZIF-8 NPs	Zn-based metal-organic framework	Passive inflammatory accumulation	Controlled Zn^2+^ release and NF-κB inhibition	M1 to M2	Preclinical	Attenuated periodontal tissue destruction	Abdelhamid (2021), Li et al. (2019b)
		PV/Ag@IL-4	IL-4 loaded polymer vesicle-silver nanoparticle	Cytokine-assisted macrophage targeting	IL-4 mediated M2 polarization	M1 to M2	Preclinical	Promoted periodontal repair and immune resolution	Li et al. (2024)
3	Acute Myocardial Infarction (AMI)	Col-Ca-NPs	Colchicine-containing nanoparticles	Passive cardiac inflammatory targeting	M2 promotion and cytokine suppression	Promote M2, inhibit M1	Preclinical	Reduced cardiac inflammation after MI	Wang et al. (2022), Liu et al. (2019), Wang et al. (2016)
		PLGA-NP	PPAR-γ agonist-loaded nanoparticle- containing pioglitazone	Cardiac macrophage targeting	PPAR-γ-mediated anti-inflammatory polarization	Promote M2	Preclinical	Improved cardiac repair and remodelling	Tokutome et al. (2019)
		Que@MOF/Man	Mannose-functionalized MOF nanoparticles	Mannose receptor-mediated targeting	ROS suppression and M2 polarization	M1 to M2	Preclinical	Enhanced post-infarction healing	Hu et al. (2024)
		TAK-242-NP	TLR4 inhibitor-loaded nanoparticle	TLR4-targeted inflammatory suppression	NF-κB inhibition	Suppress M1	Preclinical	Suppressed myocardial inflammatory injury	Fujiwara et al. (2019)
4	Atherosclerosis (AS)	KPF@MM-NPs	Kaempferol biomimetic nanoparticle	Biomimetic inflammatory site-targeting	Anti-inflammatory macrophage repolarization	M1 to M2	Preclinical	Reduced plaque inflammation and progression	Zhao et al. (2023)
		cRGD-platelet@MnO/MSN@PPARα/LXRα NPs	Platelet membrane-coated mesoporous silica nanoparticle	cRGD and platelet-mediated plaque targeting	Lipid metabolism regulation and inflammation suppression	Promote M2	Preclinical	Improved plaque regression and lipid homeostasis	Lv et al. (2025)
		HDL-mimetic NPs	High Density Lipoproteins-mimetic nanotherapeutics	Monocyte/macrophage targeting	Cholesterol efflux and anti-inflammatory polarization	M1 to M2	Preclinical	Reduced foam-cell formation and atherosclerotic burden	Zhen et al. (2024)
		PLGA-NPs	Biodegradable polymeric nanoparticles	Macrophage-targeted delivery	Macrophage reprogramming and cytokine suppression	M1 to M2	Preclinical	Promoted plaque stabilization and M2 polarization	Ma et al. (2024)
		P5c polypyridine NP	ROS-responsive polypyridine nanoplatform	Vascular inflammatory targeting	Anti-senescence and ROS reduction	Promote M2	Preclinical	Reduced senescence and vascular inflammation	Bartusik-Aebi et al. (2025)
5	Osteoarthritis (OA)	IgG/Bb@BRPL	Opsonized bilirubin Nanoparticle	Fc receptor-mediated targeting	ROS scavenging and M2 polarization	M1 to M2	Preclinical	Reduced OA progression and cartilage damage	Kou et al. (2022)
		MSN-PEI	Silica Nanoparticle	Enhanced intracellular uptake	Suppression of pro-inflammatory signaling	Suppress M1	Preclinical	Suppressed OA-associated inflammatory mediators	Shi et al. (2023)
		IgG/BRJ	Carrier-Free Bilirubin/JPH203 Nanoparticle	Fc receptor-mediated inflammatory targeting	Antioxidant-mediated macrophage repolarization	M1 to M2	Preclinical	Improved cartilage protection and inflammation control	Huang et al. (2024b)
		Ag@TiO2-NTs	Silver-loaded titanium nanotubes	Surface-mediated macrophage interaction	Anti-inflammatory polarization	M1 to M2	Preclinical	Enhanced osteochondral repair and immune regulation	Chen et al. (2020)
6	Ulcerative Colitis (UC) and Inflammatory Bowel Disease (IBD)	PSB@NP-FA	ROS-responsive, Folic Acid (FA) functionalized nanoparticle	Folate receptor targeting	ROS suppression and NF-κB inhibition	M1 to M2	Preclinical	Improved colitis symptoms and mucosal healing	Yan et al. (2023)
		Man-CUR NPs	Mannose-functionalized curcumin nanoparticle	Mannose receptor-mediated targeting	Anti-inflammatory macrophage repolarization	M1 to M2	Preclinical	Restored intestinal immune homeostasis	Han et al. (2023b)
		LBL-CO@MPDA	ROS-responsive orally-administered nanotherapeutics	Colon inflammatory microenvironment targeting	Notch/Hes1/Stat3 activation and ROS scavenging	Promote M2	Preclinical	Reduced intestinal inflammation and oxidative stress	Zhang et al. (2023)
7	Sepsis	AuNPs	Citrate-stabilized gold nanoparticles	Passive macrophage uptake	M2 polarization and cytokine suppression	M1 to M2	Preclinical	Improved survival and reduced systemic inflammation	Taratummarat et al. (2018)
		SPION-NPs	Iron-oxide-based synthetic nanoparticles	Passive macrophage uptake	Autophagy induction and IL-10 upregulation	M1 to M2	Preclinical	Protected against sepsis-induced organ injury	Xu et al. (2019)
8	Severe Acute Pancreatitis (SAP)	M-CS-E-LNC	Mannose-chitosan emodin nanocapsules	Mannose receptor targeting	CPT1-mediated lipid metabolic reprogramming	Promote M2	Preclinical	Reduced pancreatic inflammation and tissue damage	Song et al. (2024)
9	Acute Lung Injury (ALI)	PFTU@DEX-NPs	ROS-responsive dexamethasone-loaded nanoparticle	Oxidative microenvironment responsive delivery	NLRP3 inhibition and ROS scavenging	M1 to M2	Preclinical	Attenuated acute lung injury and pulmonary inflammation	Muhammad et al. (2022)
		GNP-P12	Peptide coated gold nanoparticle	Lung macrophage targeting	NF-κB inhibition	M1 to M2	Preclinical	Reduced lung inflammatory responses	Wan et al. (2020)
10	Wound healing	QCT@GNP-Ga	Gallium-modified quercetin nanoparticles	Wound microenvironment	TGF-β/Smad activation and M2 polarization	M1 to M2	Preclinical	Accelerated wound healing and tissue regeneration	Morris et al. (2014), Nanditha and Kumar (2022), Shams et al. (2022), Yang et al. (2023)
		hGM-g-PMMA NPs	Amphiphilic polymeric nanoparticles	Passive inflammatory tissue targeting	Alternative macrophage activation	M1 to M2	Preclinical	Enhanced tissue repair and inflammation resolution	Peled and Sosnik (2021)
		PEI-LBL-NC	PEI-based nanocarrier	Enhanced cellular transfection	IRF5 silencing and macrophage reprogramming	M1 to M2	Preclinical	Promoted wound closure and macrophage reprogramming	Sharifiaghdam et al. (2021)
11	Myocarditis	PSL-G-NPs	Protein-G conjugated nanomedicine	Selective macrophage targeting	Cytokine suppression and M2 polarization	M1 to M2	Preclinical	Reduced myocardial inflammation and injury	Toita et al. (2021)
12	Uveitis, Acute Kidney Injury (AKI), Parkinson’s Disease	PVP-CUR NPs	Polyvinylpyrrolidone-curcumin nanoparticle	Oxidative stress-responsive delivery	ROS scavenging and antioxidant enzyme mimicking	M1 to M2	Preclinical	Attenuated oxidative stress-associated tissue damage	Cao et al. (2024)
13	Anti-inflammation and macrophage related disorders	PU NPs	Surface functionalized polyurethane nanoparticle	Surface chemistry-mediated macrophage interaction	Autophagy induction and NLRP3/NF-κB suppression	M1 to M2	Preclinical	Suppressed chronic inflammation and foreign-body response	Morris et al. (2014), Dabare et al. (2023)

The terminology used in this table has been standardized to facilitate comparison across nanoparticle platforms. Where applicable, macrophage polarization terminology reflects the nomenclature reported in the original studies.

Abbreviations: RA: rheumatoid arthritis, AMI: acute myocardial infarction, AS: atherosclerosis, UC: ulcerative colitis, IBD: inflammatory bowel disease, SAP: severe acute pancreatitis, ALI: acute lung injury, PEG: polyethylene glycol, ROS: reactive oxygen species, NF-κb: Nuclear factor-kappa B, HIF-1α: Hypoxia-inducible factor-1 alpha, NLRP3: NOD-like receptor family pyrin domain containing 3, MOF: Metal-organic framework, MAPK: Mitogen-activated protein kinase, CPT1: Carnitine palmitoyltransferase 1, PEI: polyethyleneimine.

**FIGURE 3:**
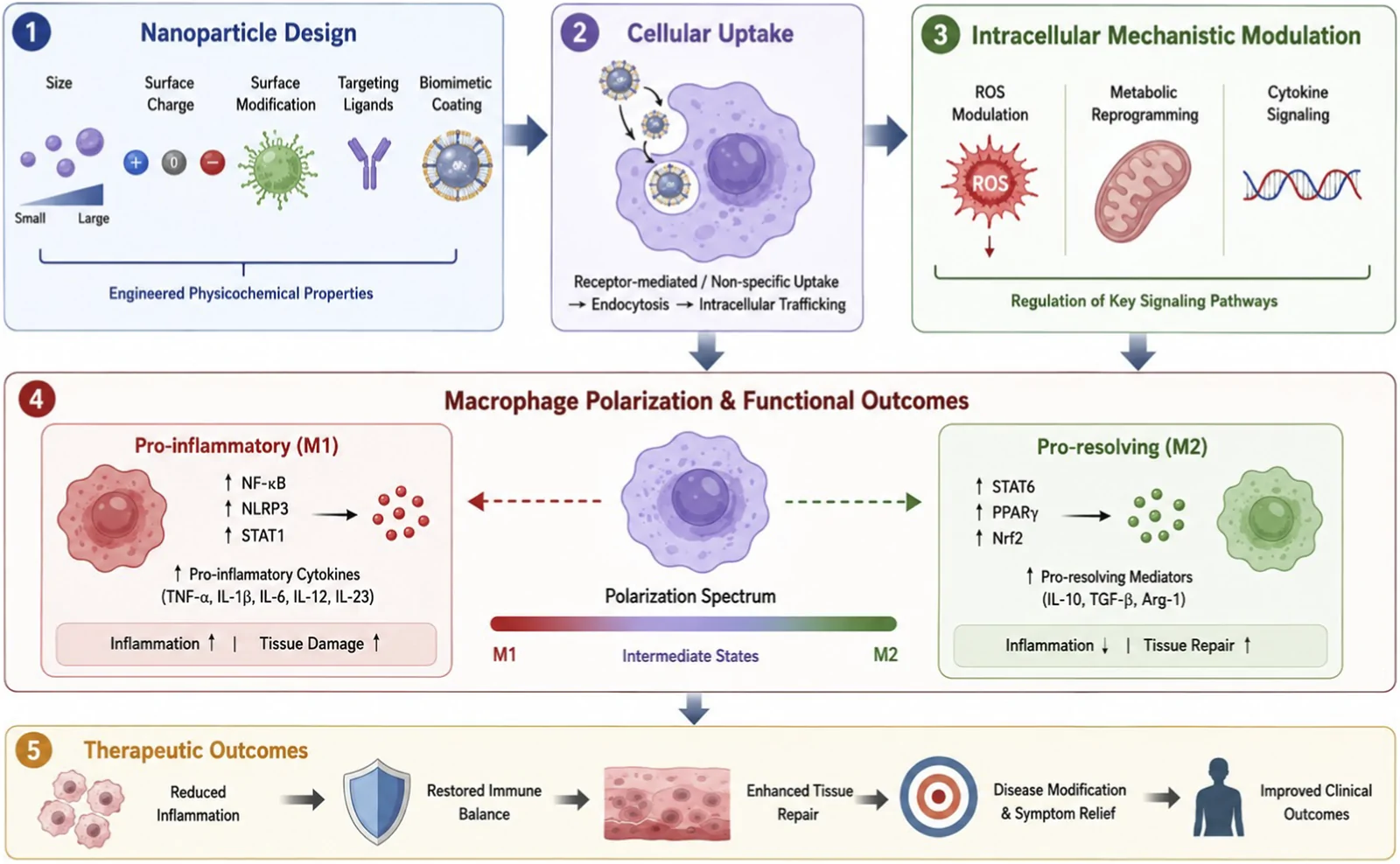
Integrated mechanistic framework illustrating how nanoparticle engineering regulates macrophage reprogramming during chronic inflammatory diseases. Nanoparticle physicochemical properties, including particle size, surface charge, ligand functionalization, biomimetic coatings, and ROS-responsive characteristics, determine macrophage uptake and intracellular signaling. These interactions regulate conserved inflammatory pathways, including NF-κB, NLRP3, HIF-1α, and oxidative stress-associated signaling, leading to context-dependent macrophage reprogramming, attenuation of inflammation, enhanced tissue repair, and improved therapeutic outcomes.

The common structure-function relationships linking nanoparticle physicochemical properties, macrophage signaling pathways, polarization outcomes, and therapeutic effects across chronic inflammatory diseases are summarized in Figure 3.

## 3 Comparative structure-activity relationships across disease models

Comparative analysis across the studies reviewed indicates that the therapeutic performance of nanoparticles depends not only on their physicochemical properties but also on the inflammatory microenvironment in which they are applied. Although common engineering strategies are employed across diseases, their effectiveness varies according to pathological characteristics and therapeutic requirements. This highlights that nanoparticle structure-activity relationships should be interpreted in a disease-specific context rather than as universal design rules.

Particle size, surface functionalization, and targeting strategies influence therapeutic outcomes differently across disease models. Smaller nanoparticles generally improve tissue penetration in diseases such as rheumatoid arthritis and inflammatory bowel disease, whereas biomimetic membrane-coated systems are particularly advantageous in vascular disorders like atherosclerosis by enhancing inflammatory-site homing and immune evasion. Similarly, ligand-functionalized nanoparticles exploit disease-associated receptor expression, with folic acid and mannose modifications improving macrophage-specific targeting in rheumatoid arthritis and inflammatory bowel disease, respectively.

ROS-responsive nanoparticles represent another common design strategy across multiple inflammatory diseases, including rheumatoid arthritis, ulcerative colitis, acute lung injury, and atherosclerosis, where oxidative stress is a major pathological driver. However, evidence from the reviewed studies suggests that multifunctional nanoplatforms integrating ROS responsiveness with active targeting or biomimetic coatings generally provide superior macrophage reprogramming and therapeutic efficacy compared with single-function systems. Collectively, these observations demonstrate that successful nanoparticle engineering relies on integrating multiple physicochemical properties according to the specific pathological features of each disease.

## 4 Cross-disease design principles, translational challenges, and knowledge gaps

Comparative analysis of the NP systems discussed in this review reveals several shared mechanistic principles governing macrophage reprogramming during chronic inflammation. Despite differences among diseases such as rheumatoid arthritis, atherosclerosis, inflammatory bowel disease, and sepsis, many successful NP platforms converge on common inflammatory pathways including NF-κB, NLRP3 inflammasome signaling, HIF-1α, STAT signaling, and oxidative stress-associated pathways (Miao et al., 2017; Li et al., 2020; Xiao et al., 2023).

Across multiple disease settings, nanoparticle-mediated macrophage reprogramming converges on a limited number of inflammatory signaling hubs. NF-κB is the most frequently targeted pathway, with several platforms suppressing pro-inflammatory cytokine production and promoting macrophage repolarization. Similarly, inhibition of NLRP3 inflammasome signaling emerges as a common mechanism underlying the therapeutic efficacy of ROS-responsive and anti-inflammatory nanoplatforms. These observations suggest that successful nanoparticle systems often act through conserved inflammatory checkpoints rather than disease-specific mechanisms alone (Guo et al., 2024; Han Z. et al., 2023; Liu et al., 2019; Zhao et al., 2023; Han X. et al., 2023Han et al., 2023b).

Another recurring theme is metabolic reprogramming of macrophages. Nanoparticles targeting hypoxia-associated HIF-1α signaling in RA and those activating AMPK–or PPAR-γ-associated pathways in inflammatory cardiovascular diseases consistently promote anti-inflammatory macrophage phenotypes. This convergence indicates that restoration of macrophage metabolic balance represents a central mechanism linking nanoparticle design with therapeutic benefit across chronic inflammatory disorders (Guo et al., 2024; Guo et al., 2021; Zhou et al., 2014; Tokutome et al., 2019). Importantly, these therapeutic effects do not necessarily reflect transition toward a single macrophage phenotype but rather the acquisition of context-dependent functional states associated with inflammation resolution and tissue repair.

Comparison across nanoparticle classes further highlights the advantages of multifunctional designs. Biomimetic membrane-coated nanoparticles improve inflammatory-site homing and immune evasion, whereas ROS-responsive systems provide controlled therapeutic release within diseased tissues. Together, these advanced platforms demonstrate how rational engineering of targeting, responsiveness, and immunomodulatory properties can enhance macrophage reprogramming and improve translational potential (Hao et al., 2024; Zhang X. et al., 2025; Zhao et al., 2023; Ma et al., 2024; Zhang et al., 2023; Muhammad et al., 2022). Collectively, these findings highlight that nanoparticle efficacy is determined not only by the therapeutic cargo but also by engineering features that govern pathway selectivity, macrophage targeting, and microenvironment responsiveness.

Importantly, comparison across studies reveals clear structure-function relationships that govern therapeutic performance. Biomimetic membrane coatings primarily enhance inflammatory-site accumulation and immune evasion, ROS-responsive materials enable selective drug release within diseased microenvironments, and ligand-functionalized nanoparticles improve macrophage-specific targeting. These design features not only influence nanoparticle biodistribution but also determine the efficiency with which key inflammatory pathways are modulated, highlighting the importance of rational engineering strategies for next-generation macrophage-directed nanotherapeutics.

Despite substantial progress, several important questions remain unresolved. First, the relationship between nanoparticle physicochemical properties and macrophage polarization remains highly context-dependent, making it difficult to establish universally applicable design principles. Second, emerging evidence suggests that macrophage activation exists along a spectrum of intermediate and context-dependent functional states rather than a simple M1/M2 dichotomy. Nevertheless, because most of the currently available nanoparticle studies continue to classify macrophages using the conventional M1/M2 framework, this review adopts the terminology used in the original publications while interpreting these findings within the broader concept of macrophage functional plasticity. In addition, the influence of protein corona formation on nanoparticle targeting, immune recognition, and therapeutic efficacy remains incompletely understood and may contribute to inconsistent outcomes across studies. Addressing these challenges will be essential for improving mechanistic understanding and advancing macrophage-targeted nanotherapeutics toward clinical translation.

Beyond these mechanistic insights, several translational barriers continue to limit clinical implementation. Although many nanoparticles’ systems successfully modulate macrophage function in preclinical studies, their clinical applicability remains limited by manufacturing complexity, variability in protein corona formation, differences between animal models and human disease, and incomplete long-term assessment. These observations underscore the need for standardized evaluation frameworks and clinically relevant validation strategies to establish robust structure-function relationships and accelerate translation.

One recurring observation is the central role of ROS modulation in controlling macrophage polarization. ROS-responsive nanoplatforms, including cerium-containing NPs, bilirubin-based systems, and antioxidant polymers, consistently reduced pro-inflammatory cytokine production while promoting pro-resolving macrophage phenotypes (Li et al., 2020; Guo et al., 2024; Zhang X. et al., 2025; Li X. et al., 2019; Muhammad et al., 2022). However, ROS scavenging alone may not provide durable therapeutic benefit, as multifunctional systems combining metabolic reprogramming, receptor-targeting ligands, or inflammasome inhibition generally demonstrated superior efficacy compared with single-function antioxidant NPs (Guo et al., 2024; Liu et al., 2019; Zhang et al., 2023).

Another important design principle is active macrophage targeting through surface engineering. Functionalization with folic acid, mannose, hyaluronic acid, antibodies, or biomimetic membrane coatings enhanced selective uptake within inflamed tissues and improved therapeutic precision (Hao et al., 2024; Qin et al., 2025; Zhou et al., 2021; Zhao et al., 2023; Ma et al., 2024). Biomimetic coatings derived from macrophage, platelet, or neutrophil membranes further improved immune evasion and inflammatory tissue homing, thereby overcoming rapid systemic clearance commonly associated with conventional nanomedicine platforms (Zhao et al., 2023; Ma et al., 2024; Bartusik-Aebi et al., 2025).

Despite encouraging preclinical outcomes, major translational barriers remain unresolved. Most studies are restricted to small-animal models, with limited evaluation of long-term biodistribution, immunotoxicity, pharmacokinetics, and large-scale manufacturing feasibility (Kumarasamy et al., 2024; Desai, 2012; Buzea et al., 2007). In addition, many reports still rely on the simplified M1/M2 macrophage classification, whereas recent transcriptomic studies demonstrate that macrophage activation exists along a dynamic spectrum of intermediate phenotypes shaped by tissue-specific microenvironments (Nahrendorf and Swirski, 2016; Li C. et al., 2019; Katkar and Ghosh, 2023; Saliba et al., 2016; Mould et al., 2019; Murray et al., 2014).

Importantly, contradictory findings across the literature indicate that NP physicochemical properties critically determine immune outcomes. While several NPs suppress inflammation and promote macrophage polarization, others may activate inflammasome pathways or exacerbate oxidative stress depending on particle composition, surface charge, dosage, and protein corona formation (Xiao et al., 2023; Martinon et al., 2006; Morishige et al., 2010; Reisetter et al., 2011). Therefore, future clinically translatable nanotherapeutics will likely require modular multifunctional designs integrating controlled ROS scavenging, disease-specific targeting, optimized biocompatibility, and standardized immune profiling approaches.

A notable limitation across the current literature is the scarcity of standardized head-to-head comparisons between nanoparticle platforms. Differences in animal models, disease regimens, administration routes, and outcome measures make direct efficacy comparisons difficult. Consequently, many apparent differences among nanoparticles systems should be interpreted cautiously until validated through systemic comparative studies.

## 5 Future perspective

Building upon the cross-disease mechanistic patterns discussed above, several NP engineering principles emerge that may guide future therapeutic development. Effective NPs usually integrate ROS-scavenging cores, precise targeting ligands, and pathway-specific payloads to restore macrophage functional balance and promote context-dependent pro-resolving phenotypes while minimizing off-target effects.

### 5.1 Core material selection

ROS-responsive or enzyme-mimetic cores, such as metal oxides in Fe-Qur NCNs, Ag-CeNPs, ZIF-8, and PFTU polymers, consistently enable broad scavenging of superoxide, H_2_0_2_, and peroxynitrite, disrupting NF-κB, HIF-1α, and NLRP3 pathways to favor pro-resolving macrophage phenotypes. Natural antioxidants like bilirubin in BSA-BR-Pt NPs or quercetin in QCT@GNP-Ga shift metabolism from glycolysis to oxidative phosphorylation via AMPK or TGF-β/Smad, offering a strategy to attenuate hypoxia-driven inflammation. Current evidence suggests that hybrid cores combining nanozyme activity with pH/ROS responsiveness may offer advantages for sustained therapeutic efficacy ensuring catalytic turnover for chronic inflammation, enabling lower doses, and reducing toxicity in multi-dose regimens.

### 5.2 Targeting strategies

Ligand conjugation with folic acid, mannose, or hyaluronic acid generally enhances selective uptake by inflammatory macrophages and may improve repolarization efficiency compared with no-targeted systems. Biomimetic coatings, including macrophage membranes (KPF@MM-NPs, PLGA-NP) and opsonization (IgG/Bb@BRPL, IgG/BRJ-NP), enhance homing to inflamed tissue via CD44 or Fc receptors, thereby reducing cytokine storms. Disease-specific ligands may improve targeting precision and pharmacokinetic performance, such as folate/mannose for joints/guts and platelet/RGD for vessels, to boost precision, drawing on the most successful NPs that use active targeting to optimize pharmacokinetics.

### 5.3 Path to clinical translation

Although all the nanoparticle platforms discussed in this review remain at the preclinical stage, their encouraging efficacy across multiple disease models highlights the therapeutic potential of macrophage-targeted nanomedicine. However, successful clinical translation will require overcoming several biological, manufacturing, and regulatory challenges. Persistent issues include scalability, long-term biocompatibility, and off-target effects, and optimization of hybrid nanoplatforms integrating ROS-scavenging cores, disease-specific targeting ligands, and pathway-specific payloads. Addressing these challenges will facilitate the development of modular nanotherapeutic platforms for macrophage-directed immunotherapy.

Despite encouraging preclinical outcomes, several important barriers continue to limit the clinical translation of macrophage-targeted nanotherapeutics. Most studies rely on small-animal models that only partially recapitulate the complexity and heterogeneity of human inflammatory diseases, limiting the prediction of clinical efficacy. In addition, long-term biosafety, biodistribution, biodegradation, and immunogenicity remain insufficiently characterized, particularly following repeated administration. Manufacturing challenges, including reproducible large-scale production, batch-to-batch consistency, quality control, and cost-effective fabrication, further hinder clinical implementation. Addressing these limitations through standardized preclinical evaluation, clinically relevant disease models, scalable manufacturing strategies, and long-term safety assessments will be essential for the successful translation.

Current evidence suggests that future nanoparticle design should be guided by context-specific structure-function relationships rather than universal engineering rules. Physicochemical properties such as particle size, surface charge, ligand functionalization, and protein corona formation collectively influence biodistribution, macrophage uptake, and polarization outcomes. While smaller nanoparticles may enhance tissue penetration and positively charged surfaces often increase cellular uptake, these effects vary substantially with nanoparticle composition, disease microenvironment, and biological interactions. Similarly, the influence of surface charge on macrophage polarization remains incompletely understood, with studies reporting context-dependent effects that may arise from differences in protein corona composition, nanoparticle chemistry, and disease microenvironment. Therefore, future rational design strategies should integrate nanoparticle physicochemical properties with disease-specific inflammatory mechanisms and macrophage phenotypes to achieve safe and effective immunomodulation (Li et al., 2020; Xiao et al., 2023; Huangfu et al., 2023; Huang et al., 2021; He et al., 2025; Dong and Wang, 2023; Zhang H. et al., 2025).

Recent advances in single-atom nanozymes (SANs) further demonstrate the expanding potential of nanoparticle engineering for macrophage reprogramming in inflammatory diseases. Owing to their atomically dispersed catalytic active sites, SANs exhibit enhanced catalytic efficiency, precise ROS regulation, and excellent biocompatibility. Emerging studies have shown that membrane-coated and microenvironment-responsive SANs effectively modulate oxidative stress, pyroptosis, and immune homeostasis, thereby promoting context-dependent macrophage reprogramming and tissue repair in models of intracerebral hemorrhage, chronic wounds, subarachnoid hemorrhage, acute myocardial infarction, and osteonecrosis. These findings highlight SANs as a promising next-generation nanotherapeutic platform with significant translational potential for chronic inflammatory diseases (Li et al., 2026; Wu et al., 2026; Hu et al., 2026; Zhu et al., 2026).

Future studies should also prioritize the resolution of conflicting observations regarding nanoparticle size, charge and corona-dependent effects on macrophage behavior. Establishing standardized experimental frameworks and clinically relevant disease models will be critical for developing predictive structure-function relationships and accelerating translation into human therapies.

## 6 Conclusion

In summary, chronic inflammation is a pivotal contributor to the pathogenesis of numerous diseases, underscoring the urgent need for innovative therapeutic approaches that restore immune balance and promote tissue repair. Targeting macrophage polarization represents a critical strategy, as the dynamic switch between pro-inflammatory and pro-resolving states governs both the progression and resolution of inflammatory responses. NPs have emerged as powerful modulators in this context, capable of precisely tuning macrophage phenotypes through mechanisms including ROS neutralization and the selective delivery of bioactive molecules. These interventions not only suppress sustained inflammatory signaling but also facilitate the restoration of vital barrier functions and microbial homeostasis, particularly within the intestinal environment. Collectively, the studies discussed in this review indicate that proresolving and ROS-scavenging NPs achieve optimal efficacy when their efficient redox activity is coupled with disease-mimetic cores, which provide broad catalytic scavenging of multiple reactive species. As research continues to elucidate the intricate crosstalk between nanomaterials and immune cells, the development of nanoparticle-based therapies holds significant promise for advancing the treatment of chronic inflammatory disorders, offering a refined and effective means to modulate inflammation at its cellular core.

While promising, challenges remain in translating NP-mediated immunomodulation into clinical practice, including ensuring biocompatibility, minimizing off-target effects, and achieving sustained therapeutic efficacy. Ultimately, integrating multidisciplinary insights from immunology, materials science, and molecular medicine will be essential to harness the full therapeutic potential of NPs in modulating macrophage-driven inflammation. The evolving landscape of nanotechnology thus holds transformative possibilities for addressing chronic inflammatory diseases that currently lack effective interventions.
